# Environmental radiological surveillance in the Baltic Sea: offshore wind farms as platforms for early-warning detection systems

**DOI:** 10.1007/s10653-026-03385-4

**Published:** 2026-08-06

**Authors:** Krzysztof Pająk, Barbara Wiaderek, Dorota Gajda, Artur Czapski, Marzena Walkowiak

**Affiliations:** 1 Maritime Operations Department, War Studies University Military Faculty, Al. Gen. A. Chruściela “Montera”103, 00-910 Warsaw, Poland; 2 Faculty of Security, Logistics and Management, Military University of Technology, Ul. Gen. S. Kaliskiego 2, 00-908 Warsaw, Poland; 3 Warsaw, Poland; 4 Military Institute of Chemistry and Radiometry, Ul. Gen. A. Chruściela “Montera” 105, 00-910 Warsaw, Poland

**Keywords:** Baltic Sea, Radiological monitoring, Offshore wind farms, Environmental surveillance, Atmospheric transport, Radiation detection, Offshore infrastructure

## Abstract

The Baltic Sea is an environmentally sensitive semi-enclosed marine basin characterized by intensive maritime activity, expanding offshore infrastructure, and the presence of nuclear-related facilities. These conditions increase the importance of environmental radiological surveillance capable of supporting the early detection of transboundary contamination events. This study assesses the potential application of offshore wind farm infrastructure as a platform for autonomous radiation monitoring within an expanded regional surveillance network.

The study combined a review of regional radiological hazards, assessment of existing monitoring infrastructure, analysis of environmental radiation data from the European Radiological Data Exchange Platform (EURDEP), and analysis of representative meteorological conditions from 2023–2024 using European Centre for Medium-Range Weather Forecasts (ECMWF) data.

The results indicate that radiological monitoring in the Baltic region is concentrated predominantly in terrestrial and coastal areas, leaving offshore regions with limited surveillance coverage. Representative atmospheric conditions indicate that airborne contaminants originating near the Gulf of Finland could potentially reach the southern Baltic coast within approximately 24 h under favourable wind conditions. Assessment of offshore infrastructure suggests that offshore wind farms could serve as platforms for autonomous radiation monitoring because they provide existing power supply, communication infrastructure, distributed offshore locations, and maintenance accessibility. Integration of offshore monitoring with terrestrial surveillance networks could improve spatial coverage and strengthen regional early-warning capability.

The proposed framework provides a basis for future atmospheric dispersion modelling and optimization of offshore radiological monitoring networks.

## Introduction

The Baltic Sea, bordered by nine countries and serving as a major corridor for maritime transport, industrial activity, and energy infrastructure, is regarded as one of the most environmentally sensitive marine regions in Europe (HELKOM, 2018). Due to its semi-enclosed character and limited water exchange with the North Sea, pollutants introduced into the Baltic environment may persist for prolonged periods and undergo relatively slow dilution processes (Fonselius & Valderrama, 2003). Historically, the region has experienced substantial anthropogenic pressure associated with industrial discharges, maritime accidents, and the atmospheric deposition of radionuclides following the Chernobyl nuclear disaster (IAEA, 2006; Ikäheimonen et al., 2009). Long-term environmental studies have demonstrated that radioactive contaminants introduced into the Baltic ecosystem may remain detectable in seawater, sediments, and marine biota for decades after contamination events, highlighting the importance of continuous radiological surveillance and environmental monitoring (Ikäheimonen et al., 2009).

Following the Chernobyl accident, cesium-137 (^137^Cs) became the dominant artificial radionuclide in the Baltic Sea. Activity concentrations in surface seawater exceeded 1,000 Bq m⁻^3^ in the most contaminated northern areas shortly after the accident, while elevated levels of strontium-90 (^90^Sr) were also detected. These radionuclides remained present in seawater, bottom sediments, and marine organisms for many years, illustrating the long-term environmental consequences of radioactive fallout (IAEA, 2006; Ikäheimonen et al., 2009).

Environmental radiological monitoring of the Baltic Sea should primarily focus on radionuclides that are relevant to routine nuclear power plant operation, potential accidental releases, and legacy contamination. Among the most important artificial radionuclides are cesium-137 (^137^Cs), strontium-90 (^90^Sr), iodine-131 (^131^I), cobalt-60 (^60^Co), tritium (^3^H), and selected transuranic radionuclides, including plutonium isotopes (^238^Pu, ^239^Pu, ^240^Pu) and americium-241 (^241^Am) (IAEA, 2005, 2006). Airborne releases associated with accidental nuclear events are dominated by volatile radionuclides such as ^131^I, ^137^Cs, and noble gases, which may be transported rapidly over long distances and deposited onto the sea surface through wet and dry atmospheric deposition. In contrast, radionuclides may also enter the Baltic Sea through riverine inflow from the extensive drainage basin, carrying contaminants originating from historical fallout, contaminated soils, industrial activities, or catchment remobilization. Their environmental behaviour differs considerably (Ikäheimonen et al., 2009). Tritium remains predominantly dissolved in seawater, while ^90^Sr behaves conservatively and is transported mainly within the water column. Cesium-137 is partly retained in seawater but is also progressively adsorbed onto suspended particles and bottom sediments. Transuranic radionuclides exhibit strong particle affinity and are therefore preferentially accumulated in bottom sediments, whereas radionuclides such as ^137^Cs and ^90^Sr may also bioaccumulate in marine organisms, particularly fish and benthic biota. These differences highlight the need for integrated environmental surveillance capable of monitoring both atmospheric deposition and marine transport pathways in order to provide early detection of radiological contamination within the Baltic Sea region (HELCOM, 2020).

The Baltic region hosts several operational nuclear power plants and is expected to undergo gradual changes in its nuclear energy sector. While existing nuclear power plants continue routine operation and modernization, some Baltic countries are evaluating the potential deployment of new nuclear technologies, particularly small modular reactors (SMRs). Although no floating nuclear power plants (FNPPs) are currently planned for the Baltic Sea, this technology has attracted international interest as a potential future option for offshore nuclear energy generation (Standring et al., 2009; Surwillo, 2022). These developments highlight the importance of maintaining effective environmental radiation monitoring systems capable of detecting accidental radioactive releases into the marine and atmospheric environment.

In addition to stationary nuclear infrastructure, the Baltic Sea is characterized by intensive maritime activity, dense commercial shipping traffic, and the periodic presence of nuclear-powered naval vessels. Although no confirmed radiological incidents related to such activities have been reported in the Baltic region, the increasing complexity of maritime operations highlights the importance of strengthening environmental early-warning systems and offshore surveillance infrastructure.

Environmental radiation monitoring constitutes a fundamental component of radiological protection and emergency preparedness. In the Baltic region, radiological surveillance is based primarily on terrestrial monitoring stations operating within national networks and supported by European and international frameworks, including the European Radiological Data Exchange Platform (EURDEP), the Helsinki Commission (HELCOM), and the International Radiation Monitoring Information System (IRMIS) of the International Atomic Energy Agency (IAEA). EURDEP enables the near real-time exchange of radiological monitoring data across Europe (De Cort et al., 2019), HELCOM coordinates regional monitoring of radioactive substances in the Baltic Sea (HELCOM, 2020), and IRMIS facilitates international information exchange on radiation monitoring capabilities and emergency preparedness (IAEA, 2020). In addition, national environmental monitoring programmes, including those coordinated by the Polish National Atomic Energy Agency (PAA) and implemented by the Central Laboratory for Radiological Protection (CLOR), provide regular measurements of radionuclides in Baltic seawater, bottom sediments, and marine biota, including cesium-137 (^137^Cs) and strontium-90 (^90^Sr) (NAEA, 2025). These programmes are based predominantly on periodic sampling of environmental media together with terrestrial radiation monitoring stations. The integration of continuous autonomous offshore radiation monitoring could complement existing monitoring networks by providing earlier detection of airborne radioactive contamination over marine areas and strengthening regional early-warning capability (De Cort et al., 2019; HELCOM, 2020; IAEA, 2020).

Meteorological and atmospheric circulation conditions over Northern Europe may facilitate relatively rapid long-range transport of airborne radioactive contaminants across the Baltic basin. Consequently, accidental radioactive releases originating from nuclear facilities located in the eastern Baltic region could potentially affect southern coastal areas within a limited time frame. This creates an important challenge for environmental monitoring systems and emergency preparedness strategies, particularly in densely populated coastal regions with extensive offshore infrastructure development.

Simultaneously, the Baltic Sea is experiencing rapid expansion of offshore wind energy infrastructure. Offshore wind farms represent large-scale, permanently powered, and spatially distributed marine platforms that may provide favorable conditions for the integration of automated environmental monitoring technologies. The incorporation of radiation detection systems into offshore wind farm infrastructure could therefore improve regional early-warning capability, enhance spatial coverage of marine radiological surveillance, and strengthen environmental and public health protection in the Baltic Sea region.

The study aims to:

(i) Assess regional sources of potential radiological risk affecting the Baltic Sea environment;
(ii) Evaluate meteorological conditions enabling atmospheric transport of radioactive contaminants toward the southern Baltic region;
(iii) Identify limitations in the current radiological monitoring infrastructure; and
(iv) Examine the technical feasibility of integrating radiation detection systems into offshore wind farm infrastructure as part of an expanded regional environmental monitoring network.

To the authors’ knowledge, the integration of offshore wind energy infrastructure with marine radiological monitoring systems in the Baltic Sea has not previously been comprehensively assessed in the context of environmental surveillance and regional radiological preparedness. The present study therefore contributes to the development of integrated offshore environmental monitoring concepts supporting marine environmental protection and transboundary radiological safety.

## Materials and methods

### Study area

The study focused on the Baltic Sea, with particular emphasis on the southern Baltic region and atmospheric transport pathways originating from the eastern Gulf of Finland. The Baltic Sea constitutes a semi-enclosed marine basin characterized by limited water exchange with the North Sea, relatively slow hydrodynamic circulation, and intensive maritime and industrial activity. The analyzed area included coastal regions surrounding the Gulf of Finland, the central Baltic Sea, and the southern Baltic coast near Cape Rozewie, Poland, which was selected as a representative endpoint for atmospheric transport assessment.

The study area was selected due to the concentration of nuclear infrastructure located within the Baltic region, Leningrad Nuclear Power Plant (Russia), Loviisa and Olkiluoto Nuclear Power Plants (Finland), and Forsmark, Oskarshamn, and Ringhals Nuclear Power Plants (Sweden), as well as expanding offshore wind energy infrastructure and existing environmental monitoring networks coordinated through European and regional radiological surveillance systems.

### Research framework

The study adopted a multidisciplinary environmental surveillance assessment approach combining:

(i) Literature-based analysis of regional radiological hazards,
(ii) Assessment of existing environmental monitoring infrastructure,
(iii) Meteorological evaluation of atmospheric transport conditions, and
(iv) Conceptual spatial analysis of offshore monitoring feasibility.

The methodological framework was designed to evaluate whether offshore wind farm infrastructure located in the southern Baltic Sea may provide suitable platforms for the expansion of regional radiological early-warning systems.

The assessment was supported by relevant scientific literature and international guidance related to environmental radiological monitoring, atmospheric transport, and environmental protection in the Baltic Sea region.

### Meteorological assessment

Meteorological conditions over the Baltic Sea during 2023–2024 were evaluated using atmospheric datasets and synoptic weather information available through the Ventusky Platform based on ECMWF (European Centre for Medium-Range Weather Forecasts) numerical weather prediction data. The assessment focused on identifying meteorological situations enabling atmospheric transport from the eastern Baltic region toward the southern Baltic coast.

Representative synoptic situations were selected based on:

- Prevailing wind direction,
- Continuity of air mass transport,
- Regional pressure distribution,
- Average wind velocity.

Atmospheric circulation events were considered relevant when air mass transport trajectories indicated potential movement from the vicinity of the Leningrad Nuclear Power Plant toward the southern Baltic Sea. Mean wind velocities along the approximate transport pathway were estimated using multiple sampling locations distributed across the Baltic basin.

Transport time was estimated using the relationship:

$$T=\frac{D}{V}$$

where

*T* = estimated transport time,

*D* = approximate transport distance,

*V* = average wind velocity along the transport pathway.

The approximate atmospheric transport distance between the Gulf of Finland region and Cape Rozewie was estimated at approximately 950 km using cartographic analysis based on Google Maps on Google Maps.

The meteorological assessment was intended to illustrate representative atmospheric transport conditions relevant to environmental surveillance planning and did not constitute a full atmospheric dispersion simulation of radionuclide transport.

### Assessment of existing radiological monitoring infrastructure

The spatial distribution of environmental radiation monitoring stations in the Baltic Sea region was evaluated using publicly available data from the European Radiological Data Exchange Platform (EURDEP). The assessment focused on identifying:

- Regional distribution of terrestrial monitoring stations,
- Offshore monitoring gaps,
- Spatial limitations in marine radiological surveillance coverage.

Particular attention was given to the southern Baltic region, where offshore monitoring infrastructure remains limited despite intensive maritime activity and expanding offshore energy development.

### Offshore wind farm infrastructure assessment

The study evaluated the feasibility of integrating radiation detection systems into offshore wind farm infrastructure as part of an expanded environmental monitoring network. The assessment considered:

- Availability of electrical power supply,
- Communication infrastructure,
- Offshore accessibility,
- Environmental exposure conditions,
- Operational continuity,
- Compatibility with automated monitoring systems.

Technical requirements for offshore radiation detection systems were analyzed based on recommendations and operational principles described in international radiation monitoring guidelines and environmental monitoring literature.

### Study limitations

The study represents a conceptual environmental surveillance assessment and does not include full atmospheric dispersion modelling, radionuclide concentration simulations, or experimental deployment of offshore radiation detectors. The meteorological analysis was limited to representative atmospheric transport conditions identified during 2023–2024 and was intended to support evaluation of regional monitoring feasibility rather than quantitative prediction of contamination levels.

Future research should include advanced atmospheric dispersion modelling, optimization of offshore detector placement, quantitative coverage analysis, and evaluation of real-time integration with regional environmental monitoring systems.

## Regional sources of radiological risk in the Baltic Sea

### Nuclear power infrastructure in the Baltic region

The Baltic Sea region contains a dense concentration of nuclear infrastructure, including operational nuclear power plants (NPPs), decommissioned reactor sites, spent nuclear fuel storage facilities, and planned future nuclear installations. Due to the semi-enclosed nature of the Baltic Sea and the relatively slow exchange of water masses with the North Sea, any accidental release of radionuclides into the marine or atmospheric environment may result in prolonged environmental persistence and transboundary contamination (HELKOM, 2018; Ikäheimonen et al., 2009). Several Baltic countries currently operate nuclear power plants located in close proximity to the coastline. Finland operates the Loviisa and Olkiluoto NPPs, while Sweden maintains operational reactors at Forsmark, Ringhals, and Oskarshamn. Russia operates the Leningrad Nuclear Power Plant near the Gulf of Finland and the Kola Nuclear Power Plant in northwestern Russia. In addition to active facilities, the Baltic region also contains decommissioned nuclear sites, including the Ignalina NPP in Lithuania and former reactor facilities in Germany (IAEA, 2025a, 2025b, 2025c). The concentration of nuclear infrastructure around the Baltic basin increases the importance of coordinated environmental radiological monitoring. Although routine radioactive releases from nuclear facilities are strictly regulated and monitored, accidental releases associated with reactor malfunction, spent fuel storage incidents, transport accidents, or external hazards remain possible (IAEA, 2006). Previous studies have shown that the long-term environmental behaviour of radionuclides in the Baltic Sea is influenced by their physicochemical properties, resulting in different transport pathways, sediment accumulation, and bioavailability within the marine ecosystem (Ikäheimonen et al., 2009). These processes should be considered when designing environmental radiological monitoring strategies. In addition to legacy contamination, future monitoring requirements will also be influenced by ongoing developments in the regional nuclear energy sector. The regional nuclear landscape is also evolving. Poland continues to implement its national nuclear energy programme through the development of its first commercial nuclear power plant at the Lubiatowo–Kopalino site. As of 2026, the project has entered the engineering and licensing phase; however, the original implementation schedule has been delayed by approximately 2–3 years, and commercial operation of the first unit is currently expected in 2036 (WNA, 2026). Estonia has selected the GE Hitachi BWRX-300 technology and is developing the legislative and regulatory framework required for its deployment, although no final investment decision has yet been made (WNN, 2025a). In contrast, Lithuania remains focused on the decommissioning of the Ignalina Nuclear Power Plant and the management of radioactive waste. Although the deployment of SMRs is under consideration, Lithuania has not yet adopted a formal implementation programme or selected a specific technology (WNN, 2025b). These developments indicate that the regional nuclear sector is gradually evolving and may significantly influence future environmental radiological monitoring requirements in the Baltic Sea region.

### Emerging nuclear technologies and offshore radiological risk

Growing interest in small modular reactors (SMRs) and floating nuclear power plants (FNPPs) introduces additional considerations for environmental radiation monitoring in marine regions. Compared with conventional large-scale nuclear power plants, SMRs are characterized by lower electrical output, modular construction, and greater deployment flexibility. FNPPs extend this concept further by locating nuclear reactors directly within marine environments (Standring et al., 2009). At present, *Akademik Lomonosov* remains the only operational floating nuclear power plant worldwide. Although deployed in the Arctic region, the technological concept demonstrates the feasibility of mobile offshore nuclear energy systems capable of operating in isolated coastal regions (Surwillo, 2022). Future deployment of similar technologies in the Baltic region cannot be excluded, particularly in areas experiencing increasing electricity demand and rapid offshore infrastructure development. The environmental significance of SMRs and floating nuclear power plants (FNPPs) is associated not only with routine operation but also with the potential consequences of accidental releases into marine and atmospheric environments. Recent safety analyses have investigated hypothetical accident scenarios, including loss-of-coolant accidents (LOCA) and severe accident conditions, demonstrating the importance of assessing radionuclide source terms, atmospheric dispersion, and potential environmental impacts in support of emergency preparedness and environmental monitoring strategies (Qiu et al., 2020). Offshore nuclear infrastructure may present unique monitoring challenges due to difficult weather conditions, limited accessibility, and the dynamic transport of contaminants through marine and atmospheric circulation systems (Standring et al., 2009). In semi-enclosed marine basins such as the Baltic Sea, radioactive contamination could persist for extended periods and affect multiple coastal states simultaneously (HELKOM, 2018). Meteorological conditions and prevailing atmospheric circulation patterns in Northern Europe may facilitate rapid transport of airborne radionuclides across the Baltic basin. Consequently, environmental early-warning systems capable of detecting elevated radiation levels over marine areas are essential for effective radiological preparedness and emergency response.

### Maritime sources of potential radiological contamination

In addition to stationary nuclear infrastructure, maritime activities constitute a potential source of radiological and environmental risk in the Baltic Sea region. The Baltic Sea is one of the world’s most heavily trafficked semi-enclosed marine basins, characterized by dense commercial shipping routes, energy infrastructure, and limited navigational space (HELKOM, 2018). Although nuclear-powered vessels are relatively uncommon in the Baltic Sea, they periodically transit through the region. The shallow depth of the Baltic Sea, narrow Danish Straits, intensive commercial traffic, and difficult navigational conditions increase the potential consequences of maritime accidents involving vessels carrying nuclear reactors or radioactive materials. In the event of a severe accident, radioactive contaminants released into the Baltic marine environment could affect seawater quality, marine ecosystems, fisheries, and coastal populations (IAEA, 2006). The environmental consequences of radiological contamination in the Baltic Sea may be amplified by the basin’s limited water exchange and slow dilution processes (Fonselius & Valderrama, 2003). Previous studies following the Chernobyl accident demonstrated that radionuclides introduced into the Baltic environment remained detectable in seawater and sediments for prolonged periods (Fonselius & Valderrama, 2003; IAEA, 2006). Consequently, even localized contamination events could result in long-term ecological and economic impacts.

### Environmental monitoring challenges associated with maritime activities

Recent years have also seen growing concern regarding poorly regulated maritime activities involving aging commercial vessels operating under opaque ownership structures and limited regulatory oversight. Such activities may complicate maritime environmental monitoring and reduce the transparency of hazardous cargo transport operations (BRAW, 2024, Kopachevsky et al., 2023). Aging vessels characterized by poor technical condition and insufficient maintenance may increase the probability of maritime accidents, including collisions, groundings, and fuel spills. Although no confirmed radiological incidents associated with such fleets have been reported, limited transparency and irregular monitoring practices may complicate the detection of unauthorized hazardous material transport or environmental contamination events (Kopachevsky et al., 2023). These factors highlight the importance of strengthening offshore environmental monitoring capabilities in the Baltic Sea. Existing radiological monitoring systems in the region rely predominantly on terrestrial measurement stations, leaving substantial marine areas with limited direct monitoring coverage. Expanding offshore monitoring infrastructure could improve early detection capacity, enhance spatial coverage of environmental surveillance systems, and support rapid response to accidental or unauthorized radioactive releases affecting the Baltic marine environment.

## Results

### Current radiological monitoring infrastructure in the Baltic Sea region

**Fig. 1:**
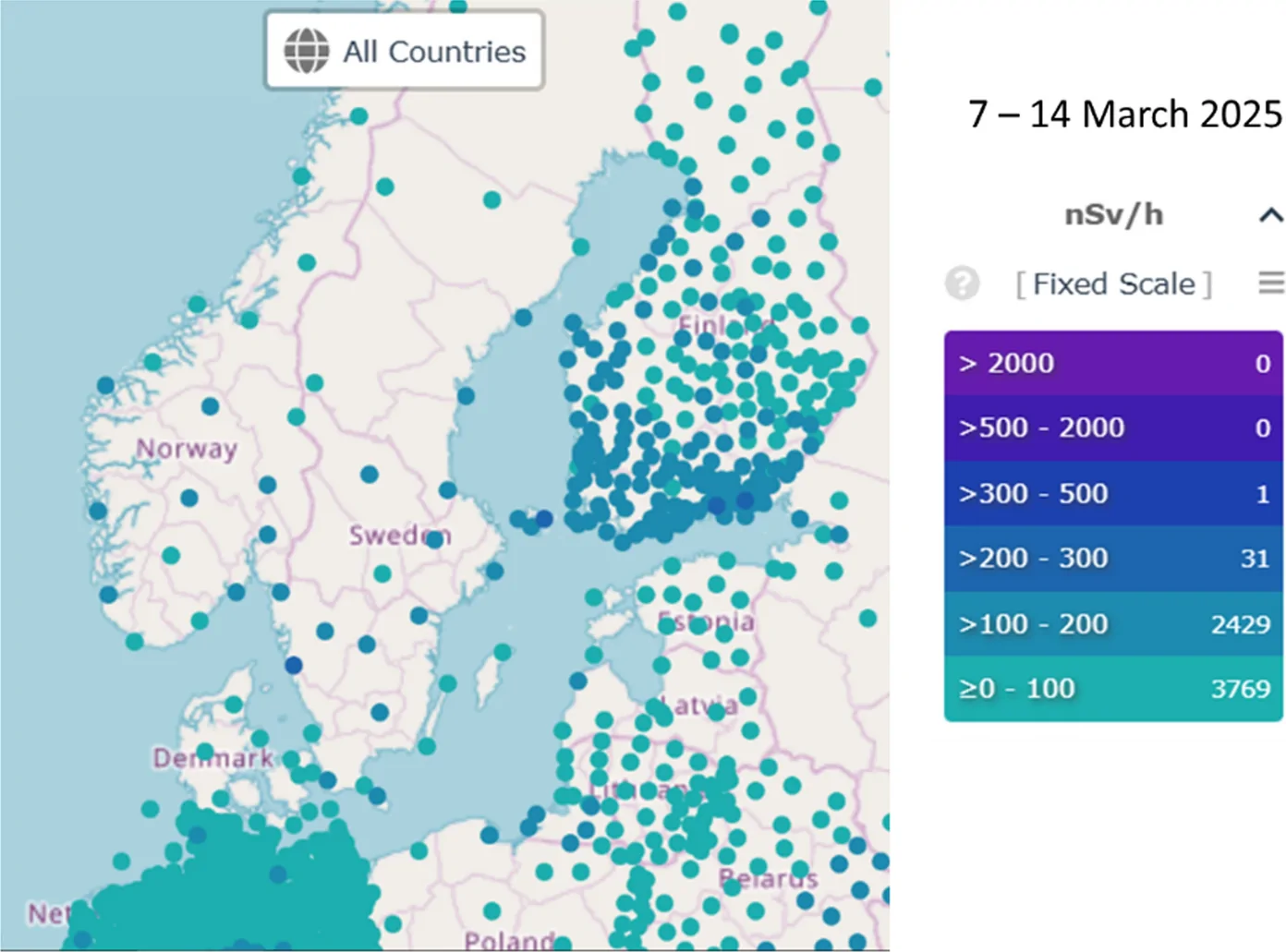
Distribution of environmental radiation monitoring stations and ambient gamma dose rate measurements in the Baltic Sea region based on EURDEP radiological monitoring data. Source: adapted from (JRC, 2025)

Environmental radiation monitoring constitutes a fundamental component of radiological protection and emergency preparedness within the Baltic Sea region. Existing monitoring systems are designed to detect variations in environmental radiation levels resulting from routine radioactive discharges, accidental releases, long-range atmospheric transport, or other radiological events affecting marine and terrestrial environments (IAEA, 2005). Monitoring activities in European Union member states are conducted in accordance with Council Directive 2013/59/EURATOM establishing basic safety standards for protection against ionizing radiation (Council of the European Union, 2014). Current environmental monitoring systems include measurements of ambient gamma dose rates and radionuclide concentrations in environmental media such as seawater, sediments, soil, and atmospheric particulate matter. Monitoring stations are distributed across national territories and in the vicinity of nuclear facilities to support rapid detection of abnormal radiation levels and transboundary contamination events. Data collected by national monitoring authorities are integrated into the European Radiological Data Exchange Platform (EURDEP), which provides near real-time radiological information across Europe (JRC, 2025). Analysis of EURDEP monitoring station distribution demonstrated that radiological monitoring infrastructure in the Baltic Sea region remains concentrated predominantly along coastal and inland areas. Although terrestrial monitoring density is relatively high in several Baltic countries, offshore regions of the Baltic Sea remain characterized by limited direct radiological surveillance coverage, particularly in southern offshore areas distant from the coastline (Fig. 1). This spatial distribution may reduce early detection capability in scenarios involving atmospheric transport of radioactive contaminants across marine environments before contaminants reach terrestrial monitoring stations.

The identified monitoring gaps are particularly relevant in the context of increasing offshore infrastructure development, intensive maritime traffic, and the concentration of nuclear facilities within the Baltic region. Existing monitoring systems were developed primarily for terrestrial environmental protection and therefore provide limited continuous surveillance over open marine areas. As a consequence, environmental early-warning capability in offshore regions may remain constrained during rapid atmospheric transport events. Table 1 summarizes the principal radiological hazard sources identified in the Baltic Sea region together with their potential release pathways, monitoring challenges, and expected environmental consequences. The overview demonstrates that the diversity of potential radiological threats requires an integrated monitoring strategy extending beyond existing terrestrial surveillance networks.

**Table 1:** Potential radiological hazard sources and monitoring implications in the Baltic Sea region

Potential radiological hazard source	Potential release pathway	Monitoring challenge	Potential environmental impact
Operational nuclear power plants (NPPs)	Atmospheric release through ventilation systems or accidental reactor incidents; liquid effluent discharge into marine or river systems	Rapid detection of low-level airborne radionuclides and transboundary contamination transport	Contamination of seawater, sediments, coastal ecosystems, and atmospheric deposition over populated areas
Spent nuclear fuel storage facilities	Accidental release associated with storage system malfunction, fire, or external disruption	Identification of delayed or low-intensity radiological anomalies in coastal regions	Long-term radionuclide accumulation in marine sediments and biota
Small modular reactors (SMRs)	Potential accidental atmospheric or liquid release during operation or transport	Limited offshore monitoring infrastructure and uncertainty related to future deployment locations	Regional marine contamination and increased environmental surveillance requirements
Floating nuclear power plants (FNPPs)	Direct offshore atmospheric or marine release associated with operational failure or maritime accident	Difficult offshore accessibility and limited marine early-warning capability	Rapid transboundary contamination affecting multiple Baltic coastal states
Nuclear-powered naval vessels	Accidental release during maritime incident, collision, grounding, or technical malfunction	Limited transparency of vessel movement and irregular offshore monitoring coverage	Localized marine contamination and environmental impact on fisheries and coastal ecosystems
Transport of radioactive materials by sea	Maritime accident involving cargo vessels transporting radioactive substances	Detection of contamination in heavily trafficked offshore transport corridors	Seawater contamination and disruption of maritime economic activities
Unauthorized or poorly regulated maritime transport activities	Potential illegal transport or unreported release of hazardous radioactive materials	Limited monitoring capability in offshore regions distant from coastal stations	Delayed detection of contamination events and reduced emergency response capability
Long-range atmospheric transport from external regions	Atmospheric transport of radionuclides across the Baltic basin under favorable meteorological conditions	Insufficient offshore detector density for early atmospheric detection	Transboundary atmospheric deposition over coastal and marine environments
Historical radioactive contamination remobilization	Resuspension of contaminated sediments during storms or dredging activities	Difficulty distinguishing historical contamination from new release events	Secondary contamination of seawater and marine food chains
Deliberate or accidental disruption of offshore infrastructure	Damage to offshore nuclear-related or maritime infrastructure during periods of regional instability	Communication disruption and reduced monitoring continuity	Local or regional radiological contamination combined with infrastructure failure

Source: author’s elaboration based on (IAEA, 2005, 2006), (HELKOM, 2018), (JRC, 2025), (Standring et al., 2009), (Surwillo, 2022), and (Kopachevsky et al., 2023).

Figure 1 presents the current distribution of environmental radiation monitoring stations and ambient gamma dose rate measurements within the Baltic Sea region based on EURDEP radiological monitoring data.

### Atmospheric transport conditions relevant to radiological surveillance

**Fig. 2:**
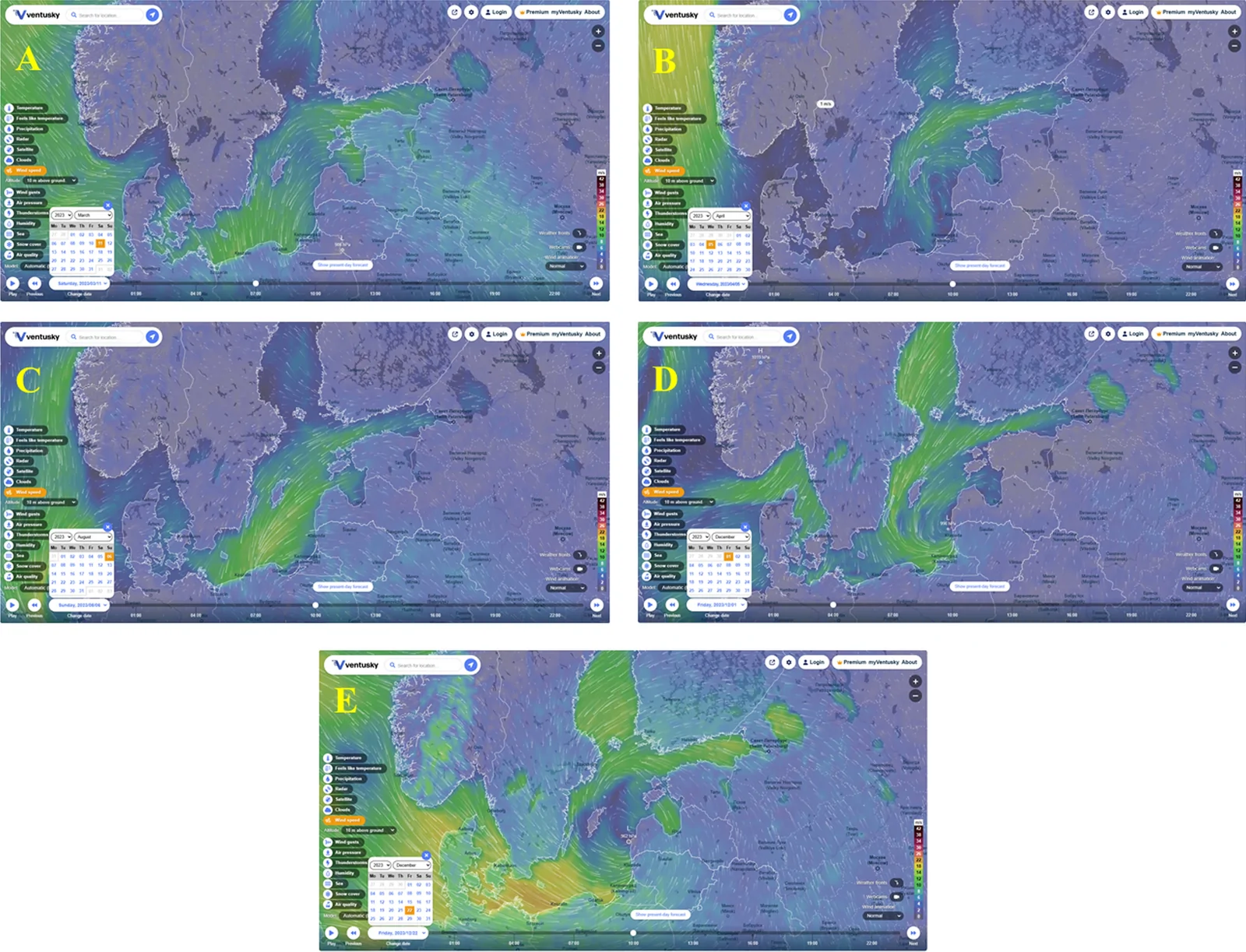
Atmospheric pressure systems over the Baltic Sea enabling air mass transport from the Gulf of Finland toward the southern Baltic region during selected meteorological situations in 2023. Source: author’s elaboration based on Ventusky meteorological datasets (2025). A – 03/11/2023, average wind speed (AWS): 9 m/s; B – 04/05/2023, AWS: 8 m/s; C – 08/06/2023, AWS: 11 m/s; D – 11/30–12/01/2023, AWS: 10 m/E– 12/22/2023, AWS: 12 m/s

**Fig. 3:**
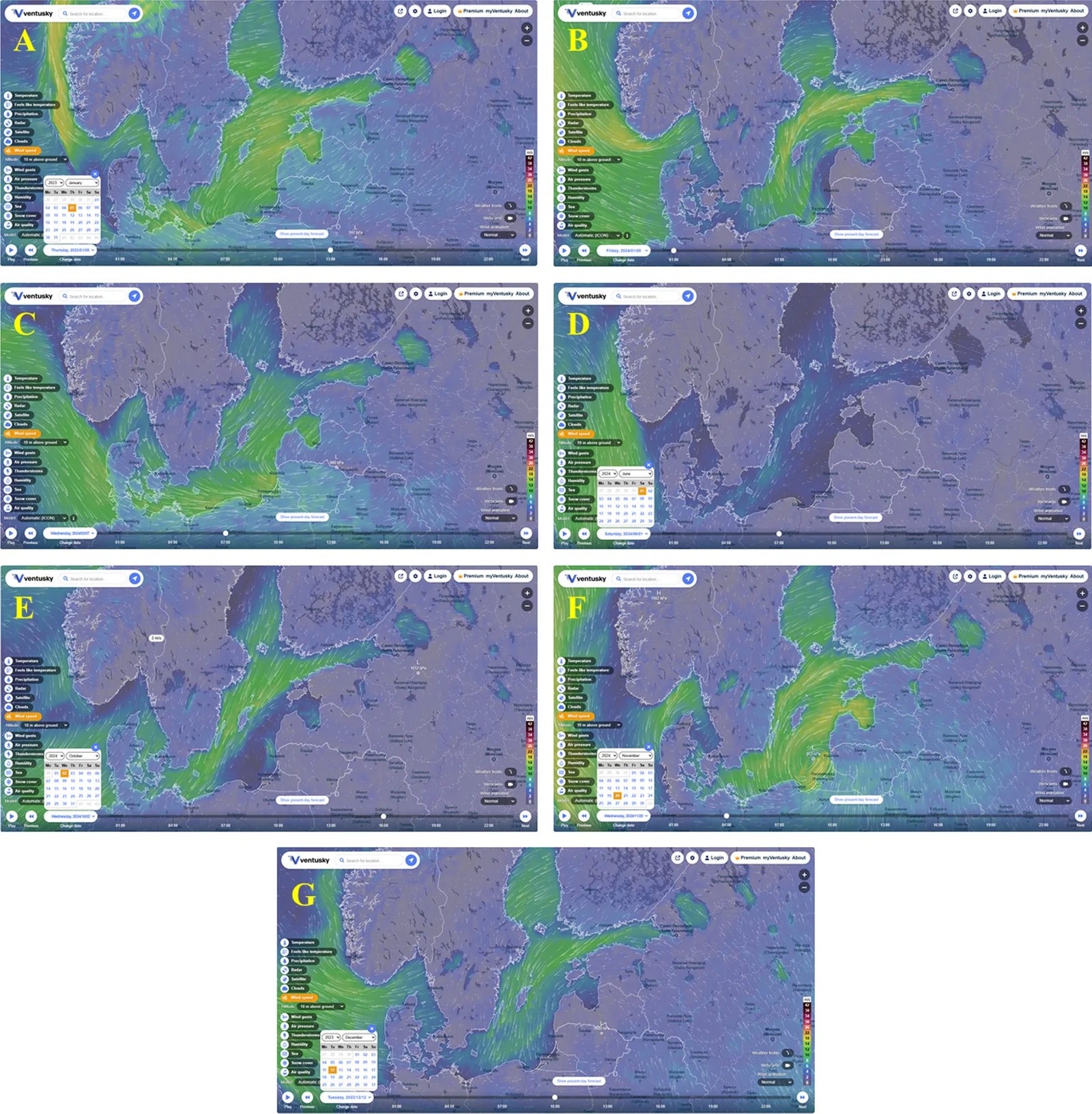
Atmospheric pressure systems over the Baltic Sea enabling air mass transport from the Gulf of Finland toward the southern Baltic region during selected meteorological situations in 2024. Source: author’s elaboration based on Ventusky meteorological datasets (2025). A – date 01/05/24, average wind speed (aws) 12 m/s; B – 01/05– 01/06/24, aws 13 m/s; C – 02/07/24, aws 10 m/s; D – 05/30–06/01/24, aws 3 m/s; E – 10/02–10/04/24, aws 12 m/s; F – 11/20/24, aws 12 m/s; G – 12/12/24, aws 10 m/s

Atmospheric circulation patterns play a critical role in determining transport pathways of airborne radioactive contaminants across the Baltic Sea region. Due to its geographical position between Atlantic and continental air masses, the Baltic basin is influenced by several dominant atmospheric pressure systems, including the Icelandic Low, the Azores High, and Eurasian continental high-pressure systems (Fonselius & Valderrama, 2003). Interactions among these systems significantly affect regional wind circulation, air mass transport, and meteorological conditions over the Baltic Sea. The meteorological assessment conducted for 2023–2024 identified multiple synoptic situations enabling atmospheric transport from the eastern Gulf of Finland toward the southern Baltic region. Representative circulation patterns were associated primarily with persistent easterly and north-easterly airflow systems extending across the central Baltic basin. Such transport conditions are particularly relevant in scenarios involving accidental radioactive releases from nuclear facilities located in the eastern Baltic region, including the Leningrad Nuclear Power Plant. Representative synoptic situations identified during the assessment are presented in Figs. 2 and 3. Wind velocities observed during the analysed meteorological situations ranged from 3 to 13 m/s, with most events occurring within the range of 8–13 m/s. The mean wind velocity calculated for the selected events was approximately 10.2 m/s.

**Fig. 4:**
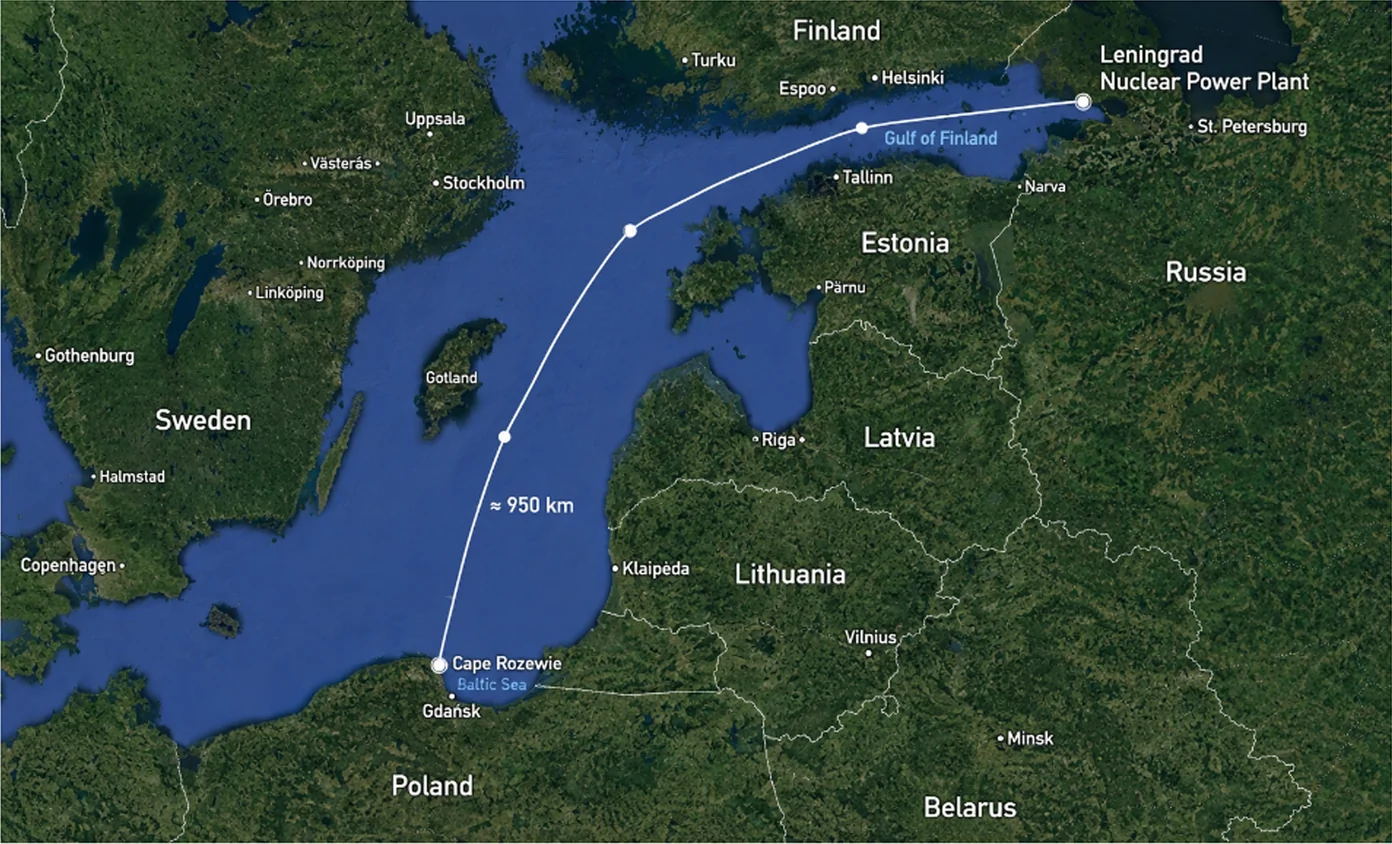
Approximate atmospheric transport trajectory and distance between the Leningrad Nuclear Power Plant and Cape Rozewie along the predominant wind circulation pathways over the Baltic Sea. Source: author’s elaboration based on Google Maps and Google My Maps cartographic data (Google 2025)

The approximate atmospheric transport distance between the Leningrad Nuclear Power Plant and Cape Rozewie along the predominant airflow pathway was estimated at approximately 950 km (Fig. 4). Assuming the mean wind velocity of approximately 10.2 m/s observed during the analysed meteorological situations, airborne contaminants could potentially reach the southern Baltic coast within approximately 24–26 h, depending on the prevailing circulation pattern.

Among the analyzed meteorological situations identified during 2023–2024, 28 circulation events enabled atmospheric transport from the Gulf of Finland toward the southern Baltic region. In 12 cases, wind conditions were sufficiently strong and persistent to allow potential long-range transport of airborne contaminants toward the Polish coast. These findings indicate that meteorological conditions favorable for transboundary atmospheric transport occur periodically within the Baltic Sea region and may significantly influence regional radiological preparedness and environmental surveillance planning.

The presented meteorological assessment was intended to illustrate representative atmospheric transport conditions relevant to environmental monitoring strategies and does not constitute a full atmospheric dispersion simulation of radionuclide transport. Nevertheless, the results indicate that offshore regions of the Baltic Sea may play an important role in future early-warning systems designed to detect airborne radioactive contamination before it reaches densely populated coastal regions.

## Discussion

### Technical feasibility of offshore radiation detection systems

The results indicate that offshore wind farm infrastructure may provide technically suitable platforms for the deployment of autonomous radiological monitoring systems in the Baltic Sea region. Existing offshore energy infrastructure possesses several characteristics favorable for environmental surveillance applications, including permanent electrical power supply, communication systems, distributed spatial coverage, and routine maintenance accessibility. Modern radiation detection systems provide broad monitoring capabilities, including ambient gamma dose rate measurements, radionuclide identification through gamma spectrometry, and detection of abnormal ionizing radiation levels above natural background conditions (IAEA, 1999). Continuous gamma dose rate monitoring represents the most operationally important function in offshore early-warning applications because it enables rapid identification of radiological anomalies associated with accidental releases or atmospheric transport events. Additional spectrometric capability may improve source identification through detection of radionuclide signatures associated with nuclear reactor incidents or radioactive contamination events. Such functionality is particularly important for distinguishing anthropogenic contamination from natural fluctuations in environmental background radiation (IAEA, 1999). The offshore marine environment imposes substantial technical and operational requirements on monitoring equipment. Offshore detectors are expected to operate continuously under conditions of high humidity, salt spray exposure, precipitation, strong winds, icing events, and large seasonal temperature variability. Consequently, detector housings and electronic components must provide high resistance to corrosion, water penetration, and mechanical degradation. The Ingress Protection (IP) classification system is commonly applied to evaluate environmental protection performance of offshore electronic systems. For marine applications, radiation monitoring devices should generally comply with protection standards ranging from IP56 to IP67, ensuring resistance to seawater exposure, dust ingress, and high-pressure water conditions. Additional durability may be achieved through the use of marine-grade anti-corrosion coatings and sealed electronic enclosures designed for long-term offshore deployment. Power demand constitutes another important operational factor. Modern dosimetric systems are generally characterized by relatively low energy consumption during standard environmental monitoring conditions. Offshore wind farms therefore represent particularly favorable deployment locations because they provide stable electrical infrastructure capable of supporting autonomous monitoring systems without the need for dedicated offshore power installations. Reliable communication between offshore detectors and centralized monitoring centers remains essential for effective environmental surveillance. Data transmission may be achieved using fiber-optic infrastructure, wireless communication systems, satellite transmission, or hybrid communication architectures. Due to the possibility of communication failure or intentional disruption, redundancy in transmission systems is recommended to ensure continuity of environmental monitoring and minimize risk of data loss. An additional operational advantage of radiation monitoring systems is their limited susceptibility to electromagnetic interference. Radiation detectors do not generate strong electromagnetic fields and generally do not interfere with offshore energy systems operating within wind farm infrastructure. Sensitive detector components, including scintillation detectors and Geiger–Müller counters, respond specifically to ionizing radiation, reducing the probability of false signals caused by environmental electromagnetic disturbances. Long-term offshore operation also requires regular maintenance, calibration, and metrological verification. According to IAEA recommendations, radiation protection monitoring instruments should undergo periodic calibration at intervals not exceeding 12 months (IAEA, 2000). Calibration procedures typically require controlled laboratory conditions and certified radioactive reference sources, necessitating temporary removal of field instruments from offshore installations. Consequently, offshore monitoring systems should incorporate maintenance strategies including detector replacement schedules, backup instrumentation, and operational redundancy. Table 2 compares the main operational characteristics of conventional terrestrial radiological monitoring systems with the proposed offshore monitoring concept. The comparison highlights the complementary role of offshore monitoring platforms in improving early-warning capability and extending surveillance coverage over marine areas.

**Table 2:** Comparison of terrestrial and offshore radiological monitoring systems in the Baltic Sea region

Parameter	Terrestrial monitoring systems	Offshore monitoring systems
Spatial marine coverage	Limited in offshore areas	Extended offshore coverage
Early atmospheric detection	Delayed for marine transport events	Improved detection capability over marine regions
Environmental exposure	Moderate	High exposure to marine conditions
Maintenance accessibility	Relatively easy	Weather-dependent offshore access
Installation infrastructure	Existing terrestrial facilities	Integration with offshore energy infrastructure
Electrical power supply	Stable terrestrial grid	Existing offshore wind farm power systems
Communication systems	Well-established terrestrial networks	Requires offshore transmission redundancy
Real-time marine surveillance	Limited	Continuous offshore environmental monitoring
Resistance to marine conditions	Standard environmental protection	Enhanced corrosion and water protection required
Operational costs	Lower maintenance complexity	Higher offshore maintenance requirements
Potential role in early-warning systems	Coastal and inland monitoring	Complementary offshore early-warning capability

Source: author’s elaboration based on (IAEA, 1999, 2000, 2005), (JRC, 2025), (HELKOM, 2018), (Delory & Pearlman, 2018), and Crise et al. (2018).

### Operational limitations and environmental challenges

Despite the technical feasibility of offshore radiological monitoring systems, several operational limitations must be considered. Marine environments create significant challenges associated with corrosion, biofouling, icing, salt deposition, and difficult offshore accessibility during adverse weather conditions. Long-term exposure to harsh marine conditions may accelerate degradation of detector housings, electronic components, and communication infrastructure. Natural variability in environmental background radiation may complicate the interpretation of low-level radiological anomalies. Variations in ambient gamma dose rate may result from changes in meteorological conditions, particularly precipitation, which enhances the deposition of naturally occurring radionuclides and atmospheric radon progeny, as well as from seasonal fluctuations in natural background radiation. Consequently, temporary increases in measured radiation levels do not necessarily indicate the presence of anthropogenic radioactive contamination and should be interpreted together with meteorological observations and radionuclide-specific measurements. Precipitation events, atmospheric radon progeny, and meteorological fluctuations may temporarily increase ambient gamma dose rates, potentially generating false-positive signals within automated monitoring systems. Consequently, offshore monitoring networks should incorporate anomaly verification algorithms and integration with meteorological datasets to improve interpretation reliability. Previous studies have identified offshore wind farms as potential targets for attacks involving unmanned aerial vehicles (UAVs), although the feasibility of such attacks depends on the technical capabilities of the UAVs and the distance of the installation from the coastline (Łukasiewicz, 2021). Consequently, future offshore radiological monitoring networks should incorporate resilience measures, including redundant communication systems, secure data transmission, and physical protection of monitoring equipment to ensure operational continuity under both accidental and intentional disruption scenarios.

Offshore wind farms constitute critical infrastructure, and the proposed concept involves integrating radiological monitoring systems with an existing critical infrastructure asset. Rather than establishing a separate offshore monitoring network, this approach could take advantage of existing power supply, communication systems, and routine maintenance operations associated with offshore wind farms. Nevertheless, the monitoring system itself should be designed as a resilient component of critical infrastructure, incorporating redundancy, cybersecurity measures, backup power supply, and operational continuity procedures to ensure reliable performance under both routine and emergency conditions (Niemi et al., 2024).

The present study did not include full atmospheric dispersion modelling or quantitative radionuclide concentration analysis. Therefore, the estimated transport times and circulation pathways should be interpreted as indicative environmental surveillance scenarios rather than predictive contamination simulations. Future studies should incorporate advanced atmospheric dispersion models, such as the Hybrid Single-Particle Lagrangian Integrated Trajectory (HYSPLIT) model or similar numerical tools, to evaluate radionuclide dispersion and deposition patterns and to optimize the placement of offshore radiation monitoring stations.

### Regional environmental surveillance implications

The identified offshore monitoring gaps and periodic occurrence of atmospheric transport conditions favorable for transboundary contamination indicate that existing terrestrial monitoring systems may provide limited early-warning capability for marine transport events affecting the southern Baltic region. Integration of offshore detection platforms with existing EURDEP infrastructure could significantly improve regional environmental surveillance capacity and strengthen radiological emergency preparedness.

The Baltic Sea constitutes a transboundary environmental system in which radiological contamination may rapidly affect multiple coastal states simultaneously. Consequently, regional radiological safety cannot rely exclusively on national terrestrial monitoring networks. Expansion of offshore monitoring infrastructure may therefore contribute to improved environmental resilience, faster anomaly detection, and enhanced coordination of transboundary emergency response activities.

The proposed integration of radiation detection systems into offshore wind farm infrastructure represents a technically feasible and environmentally relevant approach for strengthening regional radiological surveillance while utilizing existing offshore renewable energy infrastructure. Future development of integrated offshore monitoring systems may additionally support broader environmental observation capabilities through incorporation of meteorological, oceanographic, and atmospheric monitoring technologies within unified offshore environmental surveillance networks.

## Conclusions

The Baltic Sea represents a complex environmental and infrastructural system characterized by intensive maritime activity, expanding offshore energy infrastructure, and the presence of multiple nuclear-related facilities located in close proximity to marine environments. The semi-enclosed nature of the Baltic basin, combined with relatively slow water exchange processes and transboundary atmospheric circulation patterns, increases regional vulnerability to potential radiological contamination events.

Current radiological monitoring networks in the Baltic Sea region are based primarily on terrestrial monitoring stations and periodic environmental sampling, resulting in limited continuous surveillance over offshore areas. This spatial limitation may reduce early-warning capability during rapid atmospheric transport events occurring over marine environments. Based on the characteristics of offshore wind farm infrastructure reported in the literature, including the availability of power supply, communication systems, distributed offshore locations, and routine maintenance operations, offshore wind farms may provide practical platforms for the deployment of autonomous radiological monitoring systems in the Baltic Sea region.

Meteorological assessment conducted for 2023–2024 demonstrated that atmospheric circulation conditions enabling transport of airborne contaminants from the eastern Baltic region toward the southern Baltic coast occur periodically under specific synoptic conditions. Estimated transport times indicate that airborne contaminants originating near the Gulf of Finland could potentially reach southern Baltic coastal regions within approximately 24 h under favorable wind conditions. These findings emphasize the importance of strengthening offshore environmental surveillance and regional radiological preparedness systems.

The study further demonstrates that offshore wind farm infrastructure represents a technically feasible platform for deployment of autonomous radiation monitoring systems. Existing offshore energy infrastructure provides several operational advantages, including permanent electrical power supply, communication systems, distributed offshore locations, and maintenance accessibility. Integration of radiation detection technologies into offshore wind farms could therefore extend the spatial coverage of existing monitoring networks and improve environmental surveillance capability in offshore regions currently characterized by limited direct monitoring.

At the same time, implementation of offshore radiological monitoring systems requires consideration of operational and environmental challenges associated with long-term marine deployment, including corrosion, icing, communication reliability, maintenance logistics, and variability of environmental background radiation. Offshore monitoring systems should therefore be treated as complementary components of regional radiological surveillance networks rather than replacements for existing terrestrial monitoring infrastructure.

The study contributes to the development of integrated environmental surveillance concepts linking offshore renewable energy infrastructure with regional radiological protection systems. The proposed approach demonstrates that offshore renewable energy infrastructure can simultaneously support regional energy transition, environmental resilience, and radiological preparedness through deployment of distributed offshore surveillance systems.

Future research should focus on quantitative atmospheric dispersion modelling, optimization of offshore detector placement, assessment of monitoring network density, integration with real-time environmental observation systems, and evaluation of operational cost-effectiveness of offshore radiological surveillance infrastructure. Further development of integrated offshore monitoring systems may additionally support broader marine environmental monitoring and infrastructure resilience strategies within European offshore regions.

## Data Availability

No datasets were generated or analysed during the current study.
